# TikTok as an Information Hodgepodge: Evaluation of the Quality and Reliability of Genitourinary Cancers Related Content

**DOI:** 10.3389/fonc.2022.789956

**Published:** 2022-02-15

**Authors:** Xiaoqiang Xue, Xinyi Yang, Weifeng Xu, Guanghua Liu, Yi Xie, Zhigang Ji

**Affiliations:** ^1^Department of Urology, Peking Union Medical College Hospital, Chinese Academy of Medical Sciences and Peking Union Medical College, Beijing, China; ^2^Department of Health Care, Peking Union Medical College Hospital, Chinese Academy of Medical Sciences and Peking Union Medical College, Beijing, China

**Keywords:** TikTok, social media, reliability, genitourinary cancer, quality

## Abstract

**Background:**

TikTok is the world’s fastest-growing video application, with 1.6 billion users in 2021. More and more patients are searching for information on genitourinary cancers *via* TikTok. We aim to evaluate the functional quality and reliability of genitourinary cancer-related videos on it and share our thoughts based on the results for better public health promotion.

**Materials and Methods:**

We retrieved 167 videos on bladder, prostate, and kidney cancer from TikTok. Only 61 videos (36.53%) met the inclusion criteria and were eventually regarded as sample videos. Each video’s length and descriptions, hashtags, number of views/likes/comments, forms of expression, and the uploader’s profile were included. Three validated assessment instruments: the Hexagonal Radar Schema, the Health on the Net Code scale, and the DISCERN instrument, were used for evaluating the quality and reliability of the information. All misinformation was counted and categorized. Univariate analysis of variance was performed for analyzing the results. The *Post-Hoc* least significant difference test was conducted to explore further explanation.

**Results:**

Amongst 61 sample videos, healthcare practitioners contributed the most content (n = 29, 47.54%). However, 22 posts (36.07%) were misinformative, and the most common type was using outdated data. More than half of the videos could provide good (> 1 point) content on the diseases’ symptoms and examinations. However, the definition and outcomes were less addressed (tied at 21%). The HONcode scale and the DISCERN instrument revealed a consistent conclusion that most videos (n = 59, 96.72%) on TikTok were of poor to mediocre quality. Videos published by media agencies were statistically better in terms of reliability and overall score (*P* = 0.003 and 0.008, respectively). Fifty-three videos (86.89%) had at least two unexplained medical terms. Healthcare professionals tend to use professional terms most (mean = 5.28 words).

**Conclusions:**

Most videos on genitourinary cancers on TikTok are of poor to medium quality and reliability. However, videos posted by media agencies enjoyed great public attention and interaction. Medical practitioners could improve the video quality by cooperating with media agencies and avoiding unexplained terminologies.

## Introduction

Genitourinary cancer (GUCa) has posed an increasing conundrum to global Health. Crude estimates revealed 2.42 million new cases of prostate cancer, bladder cancer, and kidney cancer in 2020. Meanwhile, the yearly cancer-related death toll was reported to be 767,208 (1). Worse, less developed countries might have more trouble keeping up with the surge of cancer incidence due to the uneven development of socioeconomics (2).

The advent of social networking has offered content consumers a better experience of Web browsing, online chatting, and photo sharing, imperceptibly shifting patients’ roles from passive knowledge recipients to active information seekers (3, 4). A study based on 12,970 cancer survivors in the United States indicated that patients dissatisfied with healthcare services were more likely to search for online health information (OHI). Meanwhile, the use of the Internet for OHI acquiring had increased significantly from 2013 to 2018 (5).

TikTok, known as Douyin in mainland China, is a content-oriented social media platform that provides omnifarious genres of short-form videos. Owing to its successful team operation and low entry threshold, it has become the world’s fastest-growing social media application ever since its first launch in September 2016. Official stats disclosed that it had achieved 1.6 billion users and 2.6 billion downloads worldwide by the end of last year (6). Notwithstanding the rich and varied digital resources on TikTok, its role in healthcare promotion remains inceptive. To date, no research paper has systematically assessed the quality and solidity of GUCa content on it except for one published short communication (7).

This study attempted to evaluate the functional quality and reliability of GUCa-related videos on TikTok and offer some facts-based advice on better public health engagement.

## Materials and Methods

### Search Strategy and Data Processing

We erased all histories and settings on a smartphone to avoid potential pre-buffered cache-induced directional information recommendations. The location services were enabled while the activity tracking feature was disabled, and the language was set to Simplified Chinese by default to simulate daily life scenarios.

**Figure 1:**
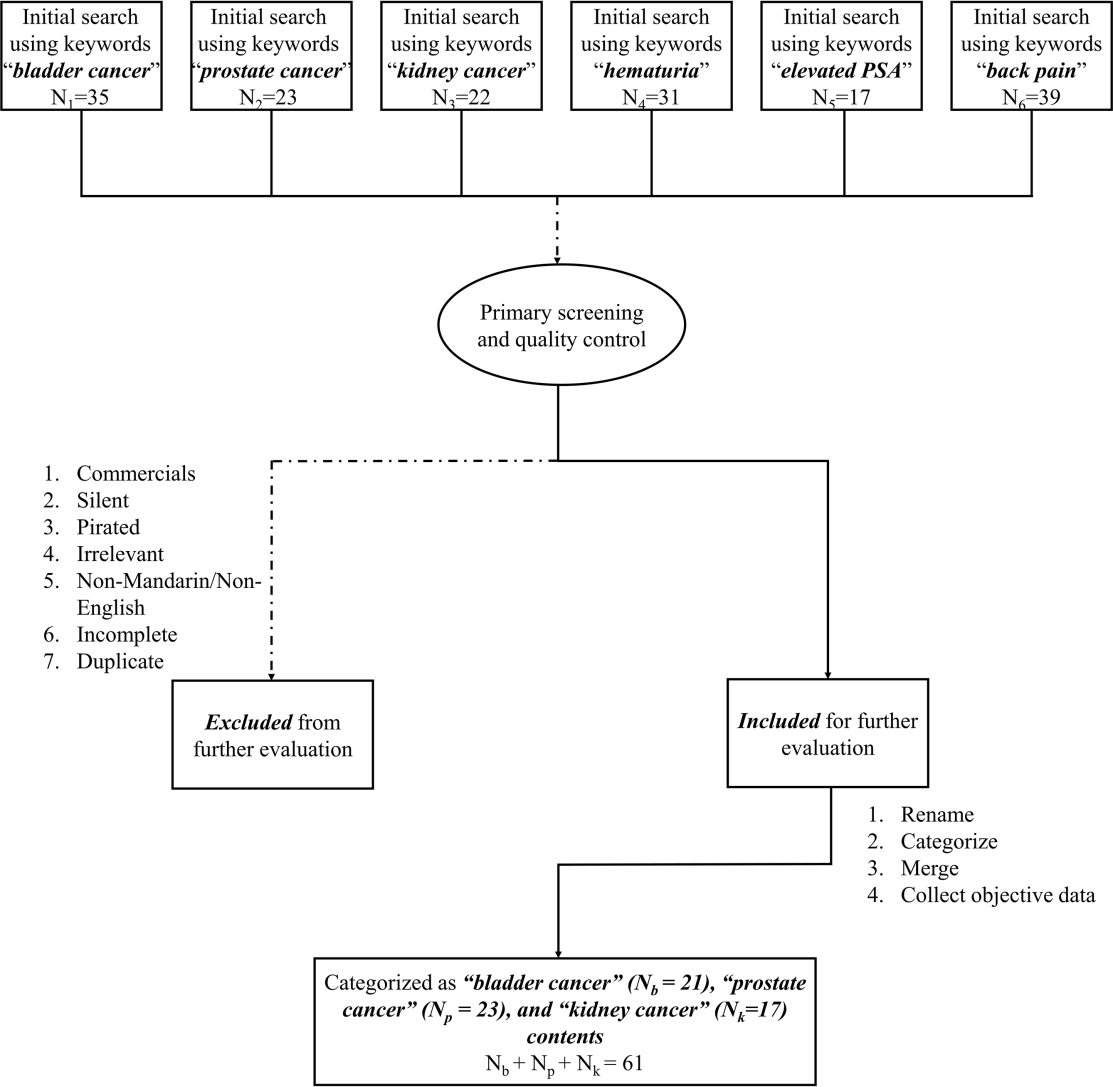
Detailed inclusion and exclusion flowchart. N_b_, Numbers of bladder cancer videos; N_p_, Numbers of prostate cancer videos; N_k_, Numbers of kidney cancer videos.

A comprehensive search was conducted on TikTok (both International and Chinese versions) from 13th to 20th September 2021. Keywords were “bladder cancer”, “prostate cancer”, “kidney cancer”, “hematuria”, “elevated PSA”, and “back pain”. To simplify the sample collecting process, the results of each keyword we used would be sorted from the most-watched to the least. All relevant videos on the first five pages of the information flow will be downloaded. As a result, a total of 167 videos were retrieved. Further evaluations were made to exclude videos with potential commercial promotions, incomplete content, linguistic barriers, or copyright disputes. It should be noted that some excluded commercial videos contained disinformation, such as exaggerating the efficacy of products and creating anxiety. After preliminary screening, 61 videos were qualified as candidates. Detailed inclusion and exclusion flowchart can be tracked in **Figure 1**. All sample videos were renamed and de-identified. Objective data were collected, including the length and descriptions of each video, their hashtags, number of views/likes/comments, forms of expression, and the uploader’s profile.

### The Definition of Quality

Since everyone could be the content creator in TikTok, we defined the “quality” as functional quality, namely, the usefulness, such as the inspiration and recommendations of the video for patients and its role in promoting health and preventing diseases. In contrast, technical quality, including video and audio signals, color and sharpness, visual effects and postediting, etc., would not be discussed due to variation in filming conditions and equipment.

### Evaluating Methodologies

In this article, we defined every downloaded video as an independent. Numbers of obvious misinformation or ambiguous knowledge points on each video were recorded, and types of misinformation would be categorized.

The HONCode is a code of conduct comprising eight procedural principles that aim to assist users in identifying the understandability, accessibility, and credibility of health information on a website (8, 9). Considering that TikTok could be visited directly through Web browsing, this code was borrowed for general assessment.

The Hexagonal Radar Schema is a coded scale that reflects six dimensions of a candidate video (i.e., the definition of the disease, signs/symptoms, risk factors, examinations, management, outcomes) (10). There would be detailed criteria and examples for reference in the schema for a specific dimension. Points from zero (not mentioned at all) to two (well-elaborated) would be given at each dimension accordingly based on the evaluation of the dimension. The spotlight and weight of each video could be visually presented by the shape and size of the radar chart. Since rationality was not a specific assessing point, all videos were accelerated in this section. A particular dimension scored more than 1 point in this chart would be regarded as acceptably clear.

The DISCERN instrument is a commonly used questionnaire for appraising the quality of written medical publications. Its robustness remains reliable in the digital era, inasmuch as it had been widely and successfully applied in rating health-related videos on YouTube, Twitter, and Facebook (11, 12). In this instrument, we mainly assessed the reliability of the content based on the following aspects: clarity, relevancy, traceability, robustness, etc. Similarly, the quality of information on treatment choices was based on the completeness of the description of the treatment methods, risks, and benefits (13).

Detailed HONCode scoring criteria, hexagonal chart entries, and the DISCERN instrument are available online as **Supplementary Material**. Two authors independently conducted all evaluations, and the verdicts were calculated as mean scores by another author.

## Results

### Characteristics of the Materials

We performed the global search using keywords and directional recommendations. The preparative appraisal excluded approximately two-thirds of the retrieved videos (n = 106, 63.47%) as they were perceived as commercial (n = 43, 40.57%), irrelevant (n = 27, 25.47%), pirated (n = 12, 11.32%), or incomplete (n = 9, 8.49%). The rest (n = 61, 36.53%) were regarded as sample videos. Among them, 57 videos (93.44%) were in Mandarin Chinese while four (6.56%) were in English.

All videos were classified into two types by defining each publisher’s username, video timeline, business model, and verified profile. Namely, videos uploaded by individuals (i.e., healthcare professionals, patients, and science communicators) or organizations (i.e., media agencies, nonprofit hospitals, and for-profit entities). Note that the patients were also seen as content creators because they did share their experiences, understandings, and advice on TikTok (n = 3, 4.92%). It was no surprise to know that healthcare practitioners contributed the most (n = 29, 47.54%), followed by media agencies (n = 19, 31.15%) and public hospitals (n = 8, 13.11%). In video length, media agencies posted the longest while nonprofit hospitals published the shortest. In general, longer videos would receive more public attention in the numbers of “likes”, “comments”, and “shares”. As the sample size of for-profit entities and science communicators was small (n = 1, 1.64%), their works were excluded in this analysis.

In terms of expression forms, media agencies were inclined to montage full television shows into episodes of video clips. In contrast, most healthcare professionals would upload videos either in a lecture-like narrative or casually captured form. The patients and their relatives tended to shoot videos with narrative complaints. Surprisingly, although videos from the patients had the lowest quality of image, stability, and audio, they were widely shared. Detailed characteristics of all sample videos are presented in **Table 1**.

**Table 1:** General characteristics of all evaluated videos.

Uploaded by (N = number of videos)	Median video length (seconds)	Median received “Likes” (times)	Median received “Comments” (pieces)	Median “Shares” (times)	Forms of expression
Healthcare professionals (N = 29)	43	1090	45	190	Narrative or shoot in OPC
Patients (N = 3)	55	2719	236	692	Storytelling
Science communicator (N = 1)	47	2329	116	437	Cartoon
Media agencies (N = 19)	87.5	2407	189	815	Clips of TV interviews
Nonprofit hospitals (N = 8)	26	754.5	14	81	Dubbing Pictures
For-profit entity (N = 1)	123	244	11	55	Conversation

TV, Television; OPC, Outpatient clinics.

### Video Content and Information Quality

Twenty-two videos (36.07%) had obvious misinformation. Some contained several errors. The most common type was the quotation of outdated data and content (n = 13, 21.31%), followed by over-interpretation (n = 5, 8.20%), the implication of supernatural or heroic power such as the self-healing of the disease (n = 3, 4.92%), blurring the differences of numbers and proportions (n = 3, 4.92%), and all-or-none thinking (thinking in an extremism way, n = 1, 1.64%).

**Figure 2:**
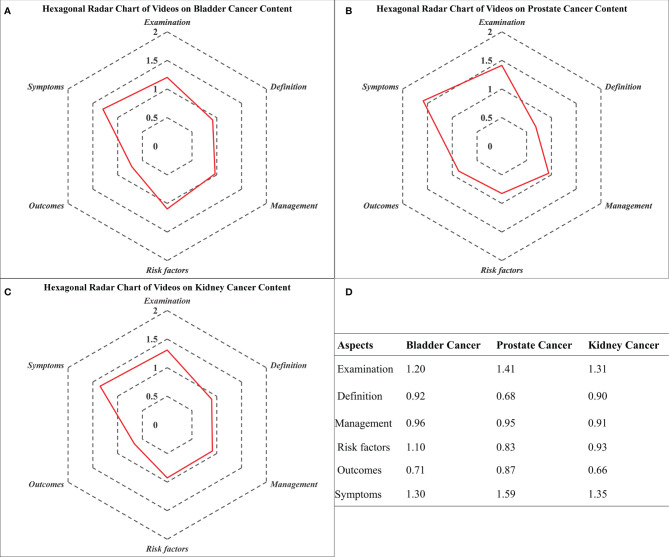
Hexagonal Radar Charts of videos on bladder, prostate, and kidney cancer content. **(A)** Hexagonal Radar Charts of videos on bladder cancer content; **(B)** Hexagonal Radar Charts of videos on prostate cancer content; **(C)** Hexagonal Radar Charts of videos on kidney cancer content; **(D)** Specific results presented as scores.

The Hexagonal Radar Charts showed the imbalance of information on TikTok. That is, more than half of the videos delivered moderate to high quality (> 1 point) content on the symptoms (n = 36, 59.02%) and examinations (n = 33, 54.10%). In contrast, the diseases’ basic definitions and outcomes were less discussed (n = 13, tied at 21.31%). On a specific disease level, the least told subjects in the bladder, prostate, and kidney cancers were outcomes (0.71 points), definition (0.68 points), and outcomes (0.66 points), respectively. Detailed scores and the Hexagonal Radar Charts are presented in **Figure 2**.

Most videos (n = 59, 96.72%) scored no more than 5 points in the HONCode scale, meaning that the quality of most videos on TikTok was somewhere between poor and mediocre. Healthcare professionals received the highest mean score of 3.76, whereas videos made by patients received the lowest of 2.5. However, the gap between the pros and amateurs showed no statistical difference.

Similarly, the DISCERN instrument suggested that the overall quality of the GUCa information on TikTok was just below moderate (2.41 out of 5). Videos published by media agencies had the highest total scores (mean score = 36.61), followed by those uploaded by healthcare professionals (mean score = 28.88) and nonprofit hospitals (mean score = 28.13). One of the reasons for the deduction was that most videos (n = 53, 86.89%) had at least two arduous medical terminologies, such as tumor heterogeneity and liquid-based cytology. Amongst them, healthcare professionals were more like to use unexplained terms (mean = 5.28 words). Videos captured by patients received the lowest quality rating scores (**Table 2**).

**Table 2:** Total DISCERN scores in different sections for all evaluated videos.

Uploaded by (N = number of videos)	Reliability of the information, mean (SD)	Quality of treatment choices, mean (SD)	Verdicts, mean (SD)	Total scores, mean (SD)
Healthcare professionals (N = 29)	17.17(3.45)	9.50 (4.20)	2.21(0.90)	28.88 (6.82)
Patients (N = 3)	13.33 (1.31)	8.33 (1.89)	2.00 (0)	23.67 (2.90)
Science communicator (N = 1)	19.00 (0)	9.00 (0)	2.50 (0)	30.50 (0)
News agencies (N = 19)	20.34 (2.80)	13.34 (7.19)	2.92 (1.02)	36.61(9.55)
Nonprofit organizations (N = 8)	16.31(5.28)	9.75 (4.49)	2.06 (1.18)	28.13 (10.62)
For-profit organization (N = 1)	24.50 (0)	7.00 (0)	2.50 (0)	34.00 (0)

SD, Standard deviation.

Further univariate analysis of variance indicated significant differences in the reliability (questions 1-8) and total scores (question 16) of videos from all uploaders (**Table 3**). The *Post-Hoc* least significant difference test indicated that the reliability and total scores were statistically higher in media agencies’ content (**Table 4**). Regarding treatment choices, TikTok offered less enchanting information. All candidates did not significantly differ, even though videos contributed by media agencies, public hospitals, and healthcare practitioners scored higher on paper (**Table 3**).

**Table 3:** The analysis of the DISCERN scores across different video sources.

Section	Type	N	Mean	Standard Deviation	F	Significance (*P*)
Question 16 (*Verdicts*)	1	29	2.207	0.911	2.585	.062
	2	3	2.000	0.000		
	4	19	2.921	1.044		
	5	8	2.063	1.266		
Question 1 to 8 (*Reliability*)	1	29	2.147	0.439	5.365	***.003***
	2	3	1.667	0.201		
	4	19	2.543	0.360		
	5	8	2.039	0.705		
Question 9 to 15 (*Treatment choices*)	1	29	1.357	0.611	2.182	.101
	2	3	1.190	0.330		
	4	19	1.906	1.056		
	5	8	1.393	0.685		
Question 1 to 16 (*Total scores*)	1	29	1.805	0.434	4.365	***.008***
	2	3	1.479	0.222		
	4	19	2.288	0.613		
	5	8	1.758	0.709		

In the Type Column, 1, Healthcare professionals; 2, Patients; 4, Media agencies; 5, Nonprofit organizations. N, number of videos.

The bold values means the values are regarded as statistically significant.

**Table 4:** The *Post-Hoc* least significant difference test for exploring the underlying differences between groups..

Section	Type		Mean Difference	Significance (*P*)
Question 1 to 8 (*Reliability*)	1	2	0.4798851	.086
		4	-0.3962114	***.004***
		5	0.1074892	.555
	2	1	-0.4798851	.086
		4	-0.8760965	***.003***
		5	-0.3723958	.23
	4	1	0.3962114	***.004***
		2	0.8760965	***.003***
		5	0.5037007	***.011***
	5	1	-0.1074892	.555
		2	0.3723958	.23
		4	-0.5037007	***.011***
Question 1 to 16 (*Total score*)	1	2	0.3257902	.319
		4	-0.4828721	***.003***
		5	0.0471444	.826
	2	1	-0.3257902	.319
		4	-0.8086623	***.018***
		5	-0.2786458	.444
	4	1	0.4828721	***.003***
		2	0.8086623	***.018***
		5	0.5300164	***.022***
	5	1	-0.0471444	.826
		2	0.2786458	.444
		4	-0.5300164	***.022***

In the Type Column, 1, Healthcare professionals; 2, Patients; 4, Media agencies; 5, Nonprofit organizations.

The bold values means the values are regarded as statistically significant.

Detailed results of the questionnaires mentioned above and charts are available as **Supplementary Material**.

## Discussion

With the popularization of smartphones and mobile Internet, social media platforms are progressively taking up more and more network traffic as banks of healthcare information for the broad public (14). However, previous data indicated that two-thirds of the online medical videos were less satisfying in quality, and nearly one-third of them were misleading (15).

The motivation and inspiration for writing this research article were that one of the authors was challenged by a patient at the outpatient clinic: “Dear doctor, I am not sure because what you have told me is different from what I learned from TikTok”.

Fode et al. reported that 71.8% of patients would search health information online. Amongst them, 14.1% indicated they would visit official healthcare websites only, whereas 85.9% admitted that they would conduct more comprehensive searches, which redirected them to other websites, including YouTube (16). Another independent survey uncovered that more than 40% of patients reported histories of quitting treatment based on advice from social media platforms (17).

Convenient as the mobile Internet was, we assumed the Pandora’s Box of online medical-related videos would interfere with traditional outpatient visits had been opened. Were the patients led astray, it could hinder the urologist-patient relationship and handicap their Health.

### Major Findings

Amongst 61 evaluated sample videos, 22 (36.07%) had unequivocal misinformation, which was in line with previous data (7, 15, 18), indicating that the correctness of online health promotion videos might not have been improved in recent years. Most misinformation was based on obsolete data and content, even at the point of time when the videos were initially posted. For instance, when discussing the treatment for prostate cancer, some videos would still recommend orchiectomy as a major method for endocrinotherapy, whereas the role of medication was less discussed, which contradicts real-world practice and triggers an atmosphere of mocking in the comments below. The rest of the misinformation randomly results from over-interpretation, confusing the concepts of numbers and proportions, and all-or-none thinking. This situation might result from TikTok’s low user barriers, as it encouraged everybody to get involved and share content as a community (19). Note that neither was TikTok’s business philosophy per se nor was the intention of most uploaders wrong. Nevertheless, considering healthcare promotion demands high professionalism and strictness, and the content had misled 40% of patients on social media to cease treating (17), allowing general users to publish highly professional content might be inappropriate.

Due to the complexity of GUCa, illustrating its diagnosis and treatment thoroughly within minutes would inevitably abridge the details. The Hexagonal Radar Charts indicated that most videos on TikTok could illuminate the symptoms and examinations of GUCa with acceptably clear quality (1.42 points and 1.37 points, respectively). Still, other aspects of the disease, such as the definition and outcomes, were lightly discussed. This result might help explain why so many outpatients take the initiative to ask for examinations after reporting their complaints, even if some are unclear whether the tumor is malignant or benign at all. A similar situation could be found in diabetes mellitus, where the particular aspect of chronic disease management was highlighted (20). This imbalance may be partly because the subjective symptoms, such as hematuria and back pain, could directly be retrieved as keywords. On the contrary, defining the disease is simple, while stating the outcomes requires more professionalism which most media agencies and science writers are not capable of. To escape this predicament, the endorsement of medical professionals into the video-producing process might be required.

This study found that 86.89% of videos on TikTok had at least two unexplained medical terms. Medical professionals used technical terms more often (mean = 5.28). As those with lower educational backgrounds and younger ages would have more difficulties understanding medical terms (21, 22), health promotion should adopt plain language. This result is higher than previous studies showing that approximately half of the videos on YouTube about prostate cancer contain undefined medical terms (16). Part of the reason might be the difference in median video length (273 seconds on YouTube versus 43 seconds on TikTok). Therefore, YouTubers might have sufficient time to define terms. However, since content creators might be likely to improvise in more time and subconsciously use medical terms, well-prepared paperwork might also be needed besides ample time.

This study ascertained that the video quality and reliability of GUCa-related content on TikTok differed with the source types. Videos published by state-owned media agencies had the highest overall quality. In comparison, those contributed by patients scored the lowest. The result is accordant with prior studies (18). Namely, media agencies are more cautious and responsible about what they have published (9). We noticed that media agencies preferred to post captioned clips of television interviews to keep the content concrete and precise. Meanwhile, sources of evidence could be given without sacrificing time. Therefore, complicated concepts were simplified, and the difference regarding the reliability and total scores was found. Strangely, videos from authoritative healthcare providers enjoyed the least popularity. A conclusion might be drawn that video length was not a factor affecting its hotness since long-form videos posted by doctors on YouTube were also the least viewed (15). Hence, we assumed the poorly designed forms of expression were to blame. As presented earlier, most healthcare professionals uploaded videos either in a lecture-like narrative or casually captured format. This identical form of expression in either short or long videos is less attractive. Previous studies based on Google searching trends and web-browsing keywords have explored OHI in various cancers. Scholars opined that the high entry threshold on reading and linguistic ability, together with the inadequacy of content, had limited the spread of OHI and sometimes misled patients (23–25). Although the advent of online video streaming has provided OHI seekers with a buffet-style array of information, we could conclude that the problem of quality and readability has been persisting across forms of OHI for many years.

The DISCERN instrument found that videos posted by media agencies were significantly better than others in terms of expressions, yet relatively weak in science and content. This might be because of their long-term experience in producing television shows. Here, we suggest that medical professionals shoot the video more vividly or directly join the media if there are technical difficulties. The content could be simplified. Still, it should be a systematic series.

### Expectations and Limitations

The role of TikTok in engaging public Health has dramatically grown during the COVID-19 pandemic (26, 27). Due to its broad user base and fast-food-style video streaming logic, TikTok is bound to become a place of strategic importance in the battle of new media. Based on current misleading information, incomplete information, and lame forms of expression, we suggest TikTok’s maximum potential to promote serious genitourinary content had not been reached. Healthcare practitioners and nonprofit hospitals could make professional content more vivid with the boost of media agencies. Meanwhile, the professionalism of media agencies’ videos could be improved with the endorsement of healthcare professionals. One is to release GUCa-related content on TikTok should be careful about the interpretation of medical terminologies, forms of expression, the focus of content, accuracy, and reliability of the information. Currently, patients should be cautious about GUCa-related information on TikTok.

Several limitations should be noted. First, most videos were in Mandarin Chinese, and a few were English. High-quality videos in other languages might be omitted. Studies on videos in other languages are welcomed, as they could contribute a more profound and comprehensive picture. Second, although the difference in the expression forms would impact the overall video quality, we did not explore the pros and cons of any specific form as currently no objective scale or questionnaire is available. Finally, because of the inequality of the professional field, no perfect solution is available to avoid misinformation on the Internet. We as healthcare professionals might feel bewildered in the face of online health information from the non-professional field. As we are both content consumers and creators in the age of mobile Internet, we would suggest the readers take the following factors into account: 1) Authority: who is the uploader? Is he a doctor or a patient; 2) Purpose: why does the uploader post this video? Does he try to recommend something, and is it exclusive? 3) Traceability: except for vague language, does the uploader provide any source of information? 4) Exaggeration does he offer unbelievable solutions or give any promises? 5) Report: repost what you find to your doctor and ask for help (28). We should expect further studies to investigate the forms of expression on the health promotion videos on TikTok.

## Conclusion

Currently, genitourinary cancer-related videos on TikTok cannot be fully trusted because some are unbalanced, ambiguous, or erroneous in content. Most of them are of poor to medium quality. To better deliver the information to the broad public, medical practitioners may need to strengthen their cooperation with media agencies and avoid turgid technical terminologies in their posts.

## Data Availability Statement

The original contributions presented in the study are included in the article/**Supplementary Material**. Further inquiries can be directed to the corresponding authors.

## Author Contributions

YX and ZJ designed the study. GL and WX collected the data. XY and XX analyzed and interpreted the data. XX drafted the manuscript. YX and ZJ supervised and gave critical revision of the manuscript for important intellectual content. XY, YX, and ZJ provided administrative, technical, and material support. All authors contributed to the article and approved the submitted version.

## Conflict of Interest

The authors declare that the research was conducted in the absence of any commercial or financial relationships that could be construed as a potential conflict of interest.

## Publisher’s Note

All claims expressed in this article are solely those of the authors and do not necessarily represent those of their affiliated organizations, or those of the publisher, the editors and the reviewers. Any product that may be evaluated in this article, or claim that may be made by its manufacturer, is not guaranteed or endorsed by the publisher.
