# Supplementary figures and images for: TikTok as an Information Hodgepodge: Evaluation of the Quality and Reliability of Genitourinary Cancers Related Content
Source: Front Oncol. 2022 Feb 15;12:789956. doi: 10.3389/fonc.2022.789956 (PMC8885733; doi:10.3389/fonc.2022.789956)

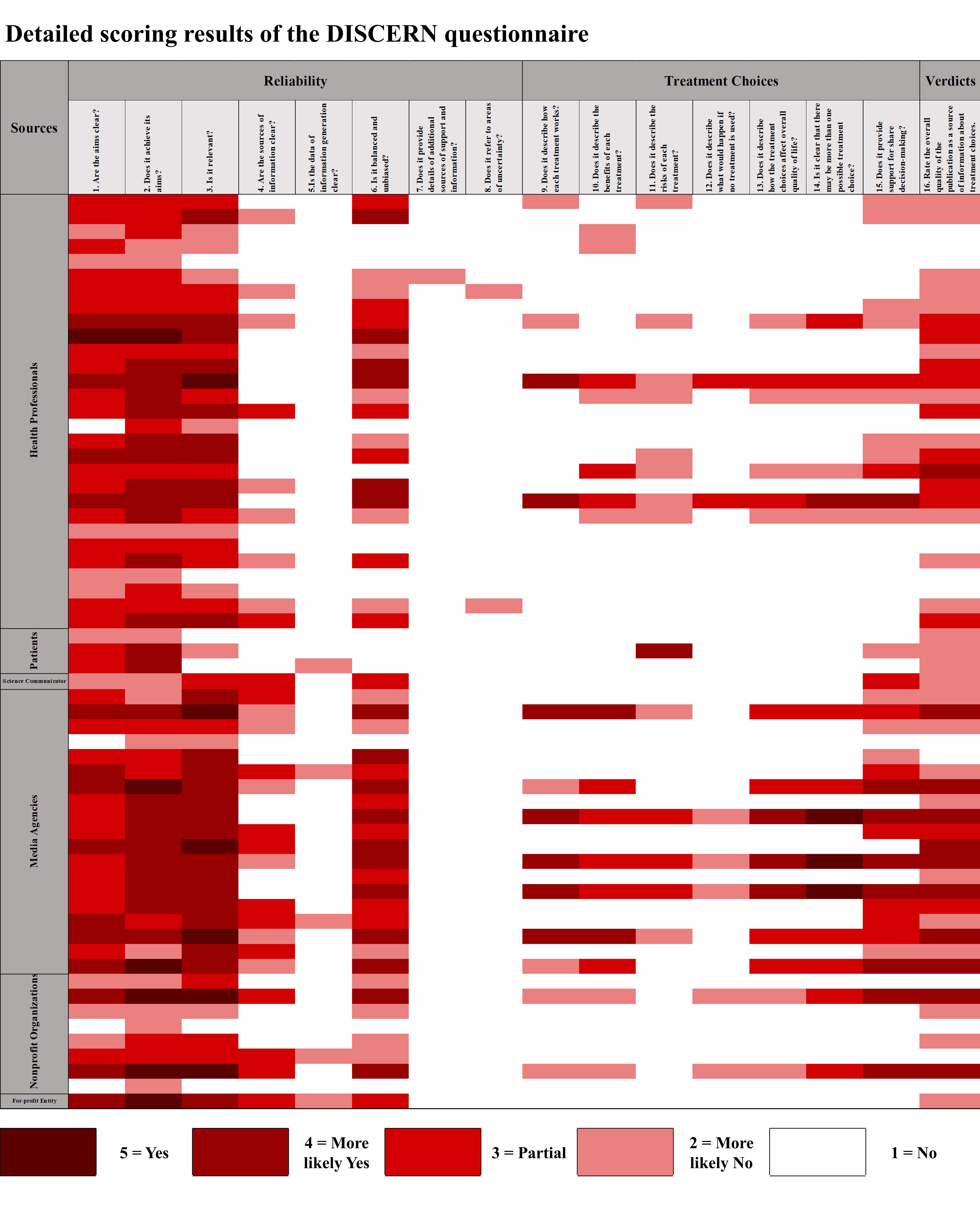

Supplement: Supplementary file 2 [file Image_2.jpeg]

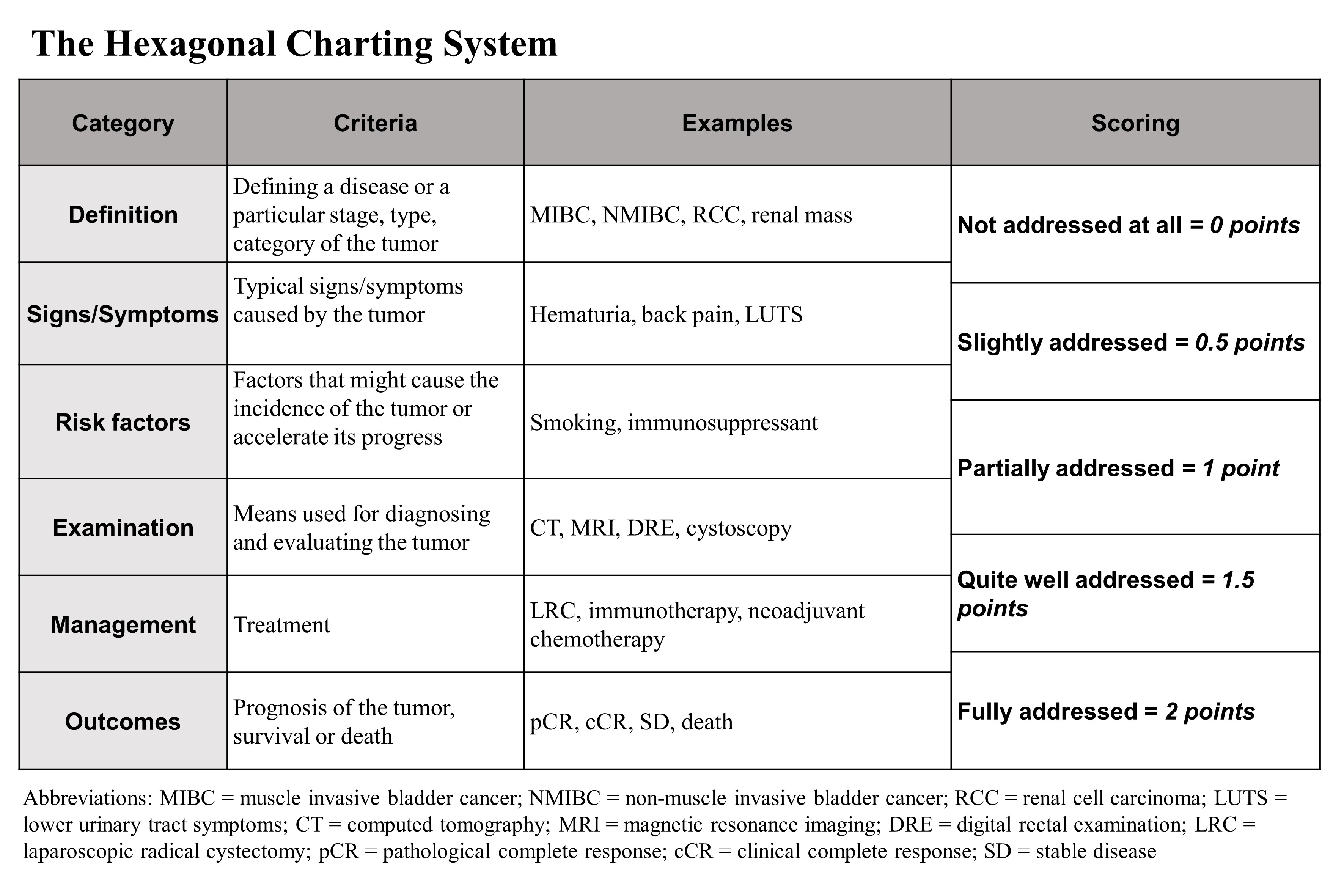

Supplement: Supplementary file 4 [file Image_4.jpeg]

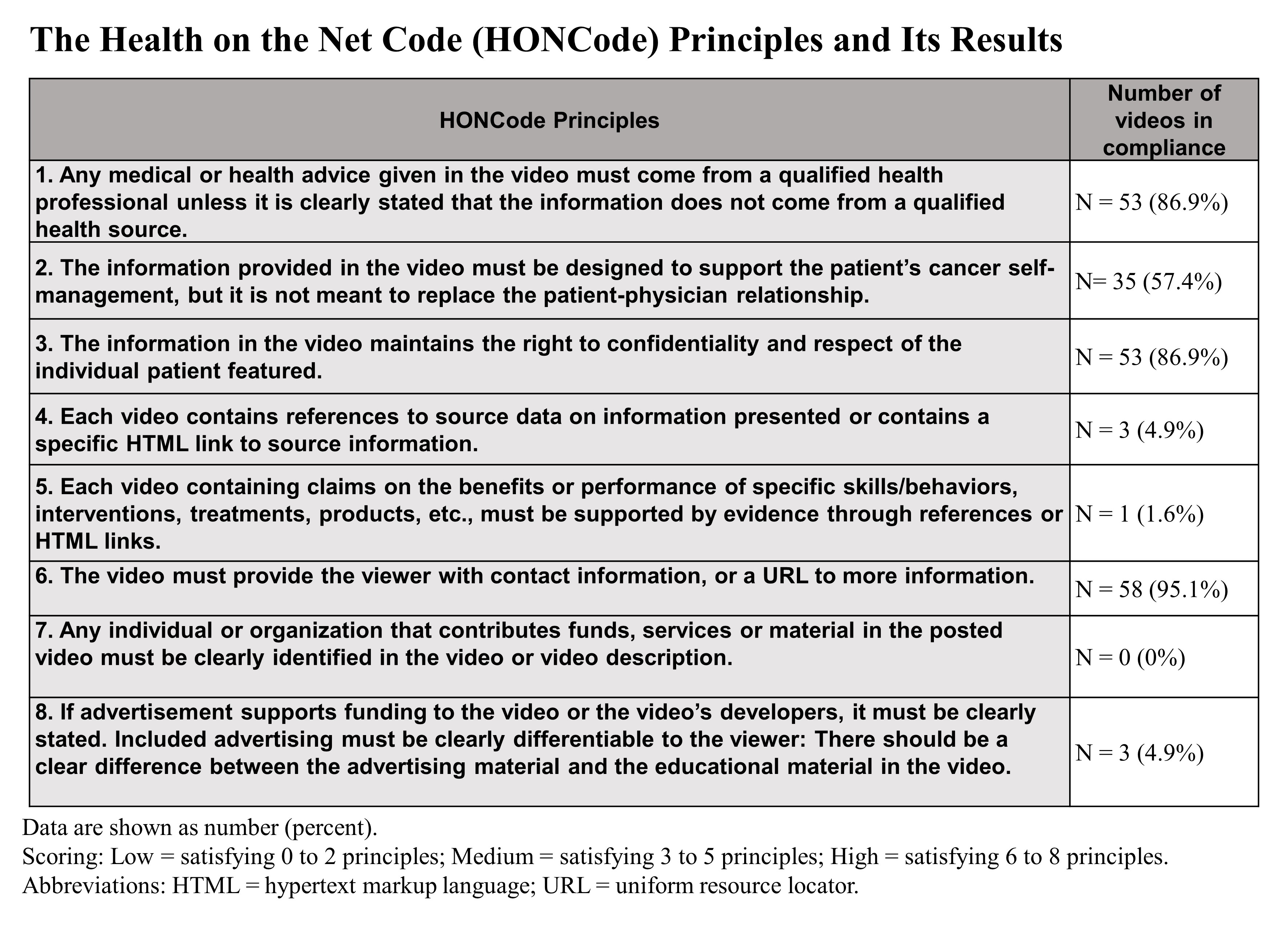

Supplement: Supplementary file 5 [file Image_5.jpeg]
